# Effect of individualized positive end-expiratory pressure based on electrical impedance tomography guidance on pulmonary ventilation distribution in patients who receive abdominal thermal perfusion chemotherapy

**DOI:** 10.3389/fmed.2023.1198720

**Published:** 2023-09-05

**Authors:** Li Xiao, Kang Yu, Jiao-Jiao Yang, Wen-Tao Liu, Lei Liu, Hui-Hui Miao, Tian-Zuo Li

**Affiliations:** ^1^Department of Anesthesiology, Beijing Shijitan Hospital, Capital Medical University, Beijing, China; ^2^Department of Science and Technology, Beijing Shijitan Hospital, Capital Medical University, Beijing, China

**Keywords:** electrical impedance tomography, individual positive end-expiratory pressure, cytoreductive surgery combined with hyperthermic intraperitoneal chemotherapy, ventilation distribution, postoperative pulmonary complications

## Abstract

**Background:**

Electrical impedance tomography (EIT) has been shown to be useful in guiding individual positive end-expiratory pressure titration for patients with mechanical ventilation. However, the appropriate positive end-expiratory pressure (PEEP) level and whether the individualized PEEP needs to be adjusted during long-term surgery (>6 h) were unknown. Meanwhile, the effect of individualized PEEP on the distribution of pulmonary ventilation in patients who receive abdominal thermoperfusion chemotherapy is unknown. The primary aim of this study was to observe the effect of EIT-guided PEEP on the distribution of pulmonary ventilation in patients undergoing cytoreductive surgery (CRS) combined with hot intraperitoneal chemotherapy (HIPEC). The secondary aim was to analyze their effect on postoperative pulmonary complications.

**Methods:**

A total of 48 patients were recruited and randomly divided into two groups, with 24 patients in each group. For the control group (group A), PEEP was set at 5 cm H_2_O, while in the EIT group (group B), individual PEEP was titrated and adjusted every 2 h with EIT guidance. Ventilation distribution, respiratory/circulation parameters, and PPC incidence were compared between the two groups.

**Results:**

The average individualized PEEP was 10.3 ± 1.5 cm H_2_O, 10.2 ± 1.6 cm H_2_O, 10.1 ± 1.8 cm H_2_O, and 9.7 ± 2.1 cm H_2_O at 5 min, 2 h, 4 h, and 6 h after tracheal intubation during CRS + HIPEC. Individualized PEEP was correlated with ventilation distribution in the regions of interest (ROI) 1 and ROI 3 at 4 h mechanical ventilation and ROI 1 at 6 h mechanical ventilation. The ventilation distribution under individualized PEEP was back-shifted for 6 h but moved to the control group’s ventral side under PEEP 5 cm H_2_O. The respiratory and circulatory function indicators were both acceptable either under individualized PEEP or PEEP 5 cm H_2_O. The incidence of total PPCs was significantly lower under individualized PEEP (66.7%) than PEEP 5 cm H_2_O (37.5%) for patients with CRS + HIPEC.

**Conclusion:**

The appropriate individualized PEEP was stable at approximately 10 cm H_2_O during 6 h for patients with CRS + HIPEC, along with better ventilation distribution and a lower total PPC incidence than the fixed PEEP of 5 cm H_2_O.

**Clinical trial registration:** identifier ChiCTR1900023897.

## Introduction

Postoperative pulmonary complications (PPCs) are common in patients after major surgery under general anesthesia with long-term machine ventilation. It is also associated with prolonged hospital stays, increased medical costs, and even higher incidences of morbidity and mortality (1). During general anesthesia, the regional ventilation distribution is impaired. Mechanical ventilation during anesthesia promotes alveolar collapse. The main pathogenic mechanism is the development of atelectasis in dorsal-dependent lung areas and overdistension in the ventral lung. As an integral part of lung protective ventilation, the setting of positive end-expiratory pressure (PEEP) plays an important role in maintaining alveolar opening, reducing alveolar shear injury, and improving gas exchange. However, too high PEEP may lead to alveolar over-dilation, increased inspiratory pressure, decreased lung compliance, impaired lung function, and adverse effects on hemodynamics (2). An international expert consensus on protective lung ventilation in surgical patients strongly recommends (3) preoperative pulmonary risk assessment with the tidal volume set at 6–8 mL/kg, positive end-expiratory pressure ventilation at 5 cm H_2_O, and pulmonary recruitment maneuvers. Moreover, among all this non-protective ventilation, PEEP is very important to prevent procedural alveolar collapse. Therefore, it is important to optimize the PEEP level individually.

**Figure 1:**
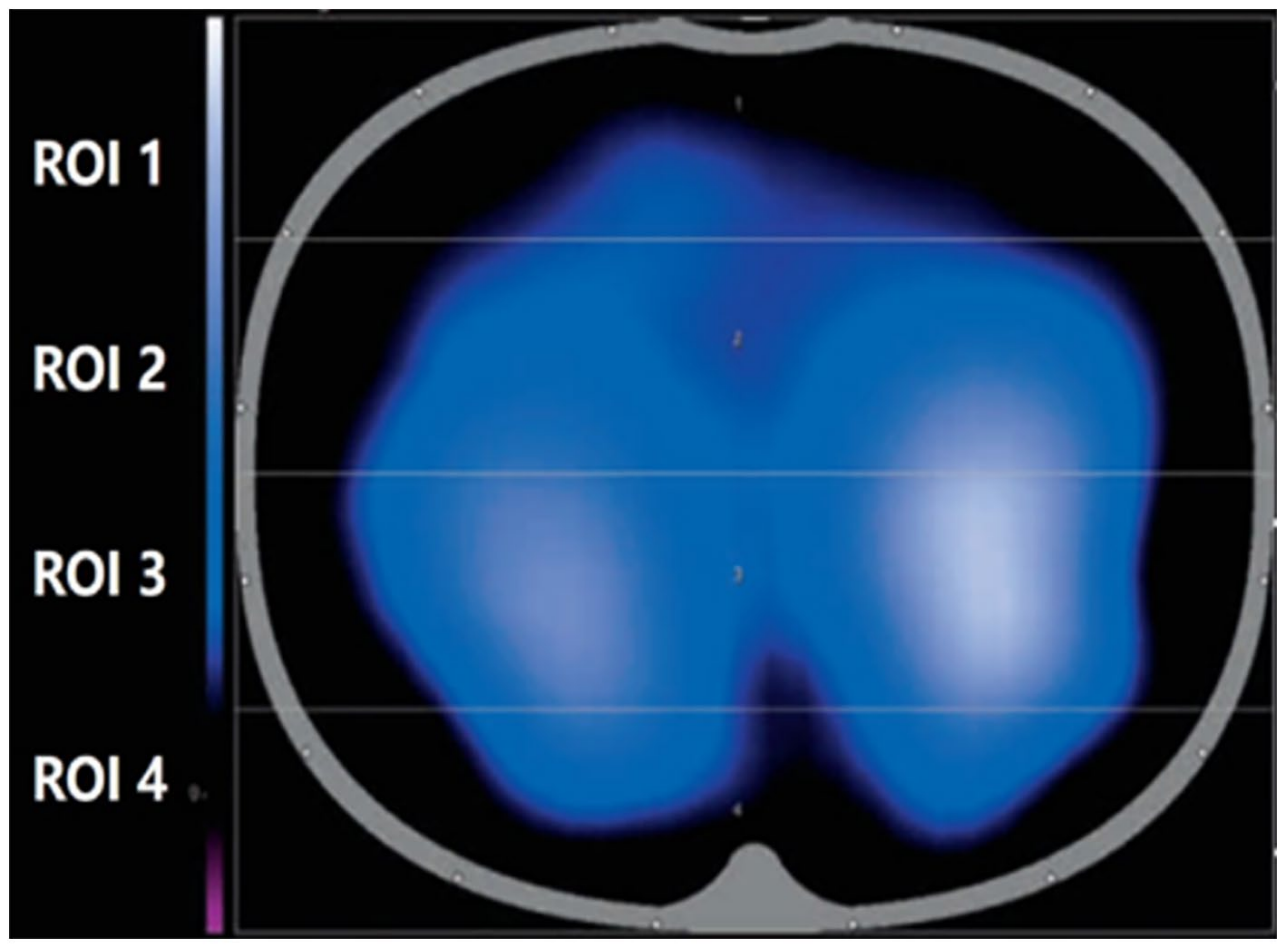
Schematic diagram of the region of interest for electrical impedance tomography.

EIT is a non-invasive, functional imaging technique that uses conductivity distribution in the human body and can be safe, portable, and dynamic in real time. When using EIT for lung monitoring, electrode sheets were placed on the chest wall, usually between the 4th and 5th ribs in the parasternal line, and a weak current was applied through local electrodes to sense changes in thoracic bioelectrical impedance during respiration. Then, the corresponding imaging algorithm was used to monitor the functional state of ventilation in different lung regions to achieve real-time dynamic presentation of tomographic ventilation images of the lung, as shown in Figure 1.

As an advanced bedside non-invasive imaging technique, EIT provides real-time mechanical ventilation monitoring. The validity of (4) EIT was confirmed through studies that stem from several previous trials, including CT (5–9), tomography (10), and MRI (11–15). EIT is characterized by the ability to perform continuous region-specific measurements of the lung in a dynamic situation. Besides, EIT was suggested to individualize PEEP to prevent atelectasis during mechanical ventilation. However, whether the PEEP level needs to be adjusted during long-term periods intraoperatively is unknown. Cytoreductive surgery (CRS) combined with hyperthermic intraperitoneal perfusion chemotherapy (HIPEC), an adjuvant therapy for abdominal malignancies, was employed in our study. CRS + HIPEC is unique in the prevention and treatment of peritoneal implantation and metastasis of abdominal malignant tumors (16–19). Owing to the long duration needed to perform laparotomy (>6 h) and mechanical ventilation, PPCs are very common in patients under CRS + HIPEC, and a lung protection strategy is essential. Therefore, the optimal PEEP level during CRS + HIPEC and the changes in the distribution of ventilation to the lungs were studied in this project.

This study investigated the optimized PEEP with long-term change by EIT for patients under major open abdominal surgery (CRS + HIPEC) because of the high risks of PPCs (17%) (20). This study aimed to apply the EIT technique to guide the titration of intraoperative individualized PEEP in HIPEC patients and to observe the effect of EIT-guided PEEP on the distribution of pulmonary ventilation and postoperative pulmonary complications.

## Materials and methods

### Participants

This study was approved by the ethics committee of Beijing Shijitan Hospital, Capital Medical University, and registered in the Chinese Clinical Trial Registry (ChiCTR1900023897) (17 June 2019). This experiment is a single-blind, randomized, controlled trial. All patients provided informed consent to confirm that all studies were conducted in accordance with relevant guidelines/regulations and that informed consent was obtained from all participants. A total of 48 patients who underwent elective CRS + HIPEC for peritoneal cancer from January 2020 to December 2021 were recruited and randomly divided into two groups, with 24 cases in each group. The inclusion criteria were as follows: (1) age over 18 years and less than 80 years and (2) undergoing surgical procedures with durations exceeding 6 h. Conversely, the exclusion criteria were as follows: (1) body mass index (BMI) >30 kg/m^2^; (2) individuals with acute or chronic respiratory conditions or a recent history of using β2 receptor agonists, hormones, ipratropium bromide inhalation, or theophylline in the past 2 weeks; (3) those with permanent or temporary cardiac pacemakers, implantable cardiac defibrillators, and other active implants; (4) those with moderate or large pleural effusion; and (5) those with severe hemodynamic instability (need the vasoactive drugs before operations). The subjects were excluded from the clinical study when the following conditions occurred: (1) pulse oxygen saturation <92%; (2) the maximum end-inspiratory pressure during mechanical ventilation was greater than 40 cm H_2_O; (3) the bleeding volume was greater than 1,500 mL; and (4) other situations that threaten the life of a patient and require rescue.

### PEEP titration strategy

A titration of EIT was performed after induction. The principles of the titration method were as follows: (1) maintain a certain depth of anesthesia to avoid choking responses and hemodynamic fluctuations during recruitment maneuvers due to too-light anesthesia; (2) the recruitment maneuver must be performed before titration begins; (3) the driving pressure (driving pressure = *P*_plat_ − PEEP) was kept constant; and (4) if it was found that the PEEP value that could maintain a stable and constant end-expiratory impedance was not unique, the smaller value was selected as the best PEEP.

Recruitment maneuver refers to the intermittent administration of higher pressure than conventional ventilation during mechanical ventilation and its maintenance for a certain period to reopen collapsed alveoli in order to improve oxygenation and reduce lung injury. After increasing the depth of anesthesia appropriately, a recruitment maneuver was performed (pressure of 30 cm H_2_O for 40 s).

In this study, titration was performed using the stable EIT-EELI method. Based on the approximate range of individualized positive end-expiratory pressure titrated from the pre-experiment, PEEP was first set at 16 cm H_2_O for ventilation, followed by stepwise changes in PEEP at 14, 12, 10, 8, and 6 cm H_2_O levels, and changes in the slope shown by EIT were monitored. PEEP optimization was performed by adjusting PEEP to obtain a horizontal EIT baseline by stabilizing end-expiratory volume. See Supplementary Figure S1. In the end-expiratory impedance images, the PEEP value that maintains a constant end-expiratory lung impedance (without progressive decrease) was selected as the most optimized respiratory parameter index, i.e., the optimal PEEP intraoperative recruitment maneuver and EIT titration are performed every 2 h in the experimental group.

### Anesthesia and ventilation protocol

After entering the room, we established venous access and routinely monitored BP, HR, ECG, SpO_2_, PEtCO_2_, and BIS. Radial artery and internal jugular vein catheterization were performed under local anesthesia to monitor mean arterial pressure (MAP), cardiac index (CI), and Stroke volume variability (SVV) for goal-directed fluid therapy. All patients were mechanically ventilated using the same anesthesia machine (MAQUET FLOW-i). The fresh air flow was 2.0 L/min, and the inspired oxygen concentration (FiO_2_) was set to 50%. The patients were laid in the supine position. After intravenous induction with midazolam 0.06 mg/kg, rocuronium 0.6 mg/kg, target-controlled infusion of propofol 3 μg/mL, remifentanil 8 μg/mL, and local mucosal surface anesthesia, an endotracheal tube was placed, and the patient was ventilated in a capacity-controlled ventilation mode. The fresh gas flow rate was set at 2 L/min and the inspired oxygen concentration (FiO_2_) at 50%. Muscle relaxants (rocuronium bromide) were given intermittently. Sevoflurane or propofol with remifentanil was used for anesthesia maintenance to maintain the bispectral index (BIS) between 40 and 60. Goal-directed fluid therapy was implemented for intraoperative fluid management. The anesthesia machine’s respiratory monitoring system monitors the respiratory parameters at each time point through the bypass airflow method. The tidal volume was set to 7 mL/kg based on the ideal body weight. The respiratory rate was adjusted according to the end-tidal partial pressure of carbon dioxide (PETCO_2_) at 35–45 mmHg, and the inspiratory ratio was configured at *I*:*E* = 1:2. Subjects were excluded from this clinical study when the following conditions occurred: pulse oximetry (SpO_2_ <92%); peak airway pressure ≥40 cm H_2_O during intraoperative mechanical ventilation; severe hemodynamic instability caused by the administration of positive end-expiratory pressure (PEEP); bleeding greater than 1,500 mL; or other conditions that posed a threat to the patient’s life safety and required the immediate suspension of the operation and emergency resuscitation.

PEEP in the control group (group A) was set to 5 cm H_2_O as the clinical routine in our institution for this kind of surgery. Intraoperative recruitment maneuvers were also performed every 2 h in the control group. Group B was the EIT (Dräger PulmoVista^®^ 500, Germany)-guided individualized PEEP. Volume control in constant flow mode was used in both groups of patients. In group B, the 16 electrical levels of EIT were evenly attached to the chest wall at the level of the patient’s xiphoid process, and the reference electrical level was located on the right chest. Electrical impedance tomography was used to monitor lung and respiratory movements in real time. The obtained electrical impedance tomography region of interest (ROI) was divided into four equal parts from the ventral side to the dorsal side and divided into four parts, namely ROI 1, ROI 2, ROI 3, and ROI 4. The ROI 3 and ROI 4 regions were considered dorsal gravity-dependent lung areas, while the ROI 1 and ROI 2 were ventral non-dependent lung areas. PEEP titration was performed as reported following the instructions at each time point (21). We designated the following time points in relation to tracheal intubation: 5 min as T1, 2 h as T2, 4 h as T3, and 6 h as T4. T0 was the period with spontaneous breathing before anesthesia induction. At each time point, the radial artery was taken for blood gas analysis. The following data were calculated and collected: PaO_2_/FiO_2_. Pulmonary dynamic compliance was recorded from the anesthesia machine; it is the pulmonary compliance measured during the respiratory cycle when airflow is not blocked and is influenced by both lung tissue elasticity and airway resistance. Postoperative pulmonary complications in patients were diagnosed by x-ray results, usually performed on day 7 after surgery. Postoperative pulmonary complications were referred and, according to the 2018 standardized perioperative care endpoints, met any of the following: (1) imaging-confirmed pulmonary atelectasis; (2) pneumonia (using centers for disease control criteria); (3) adult respiratory distress syndrome (using the Berlin consensus definition); and (4) aspiration pneumonia (clear clinical history and imaging evidence) (22). The differences between the two groups were compared.

Randomization was performed using a randomization system generated by the STATA software. Random sequences were hidden in numbered, opaque envelopes. After obtaining consent, the researchers opened random envelopes to reveal the patients’ groups. The patients were blinded to the allocation. The EIT and PEEP settings were performed by the anesthetist, not the radiologist. Due to the particularity of the intervention, the EIT intervention anesthesia was not blind to treatment allocation. Postoperative follow-up was conducted by a professional staff who did not know the design or purpose of the trial and collected the information after surgery. Data processing personnel do not know the process and purpose of the trial design.

### Statistical methods

Our primary outcome indicator was the difference in patient pulmonary ventilation function between the two PEEP regimens, and a secondary outcome indicator was the difference in the incidence of postoperative PPCs between the two groups.

The sample size was calculated based on the difference in ventilation distribution in ROI 3 and ROI 4 between groups. According to our preliminary results and a previously reported study (23), we found out that 22 patients would be enrolled to obtain a 10% difference between groups with an alpha level of 0.05 and an SD of 10%. Considering a 10% dropout rate, 24 patients were planned to enroll and to be analyzed in each group.

The experimental data were analyzed and tested using GraphPad Prism 7.0 statistical software, and the measurement data were expressed as mean ± standard error (*x* ± *s*). Independent *t*-tests or the Mann–Whitney *U*-tests were used for comparisons between groups. The Tukey test was used for *post-hoc* comparisons. The chi-squared test was used to compare categorical data. *p*-values less than 0.05 were considered statistically significant. Pearson’s product-moment correlation coefficient test was used for correlation.

## Results

**Figure 2:**
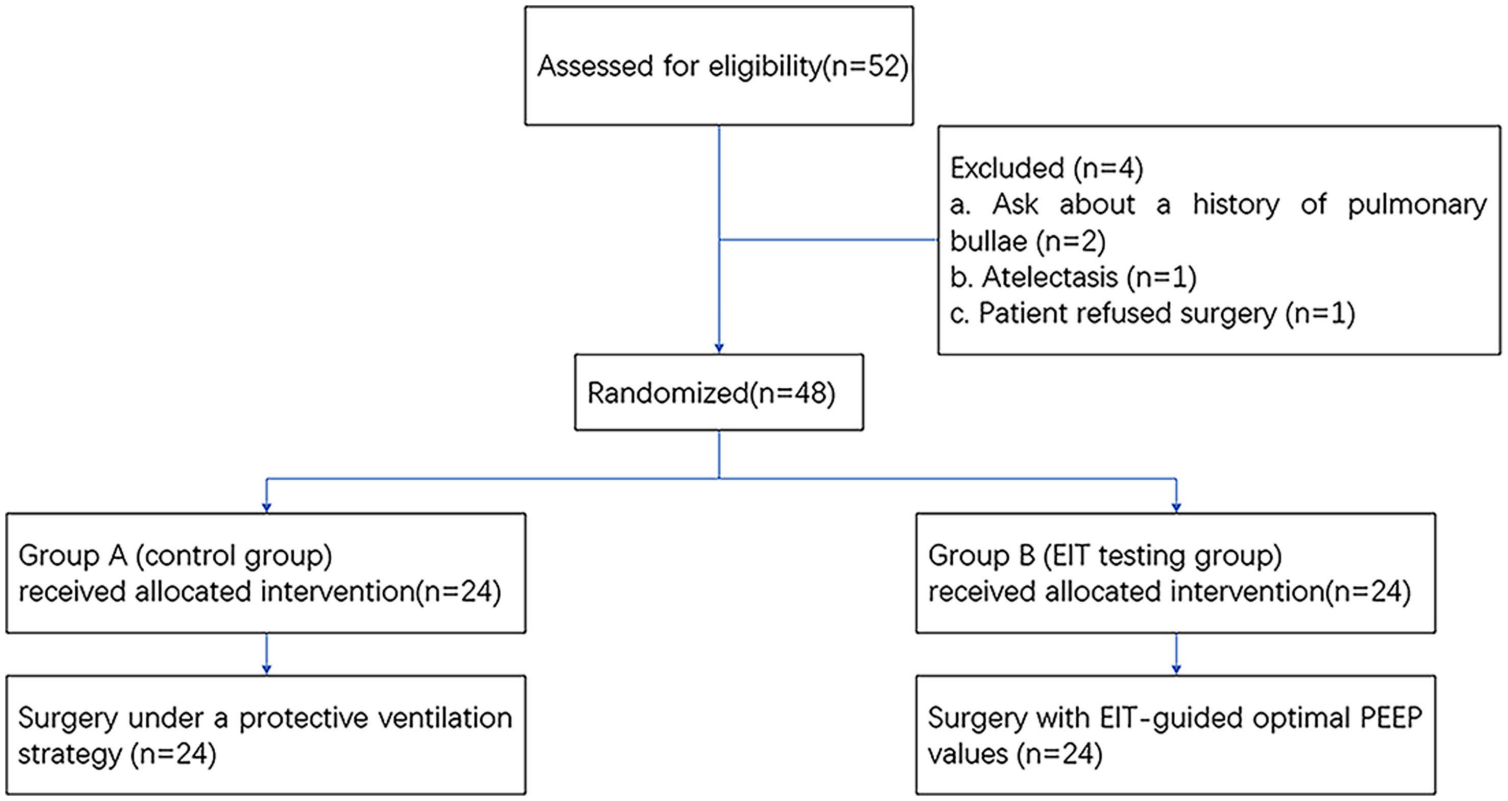
Flowchart of enrollment and outcomes. EIT, electrical impedance tomography; PEEP, positive end-expiratory pressure.

As shown in Figure 2, a total of 52 subjects were offered the intervention, and finally, 48 patients were enrolled in the study according to the original study protocol (24 in group A; 24 in group B). Four patients were excluded for refusing surgery (*n* = 2) and making the decision to withdraw from the study (*n* = 2). We analyzed differences between patients’ clinical characteristics, including age, sex, BMI, ASA classification, duration of surgery, duration of anesthesia, crystal liquid, colloidal liquid, blood, plasma, and RBC transfusion volumes, the dosage of norepinephrine (NE), urine, total fluid input, and output. These indicators were not found to be statistically significant (*p* > 0.05). See Table 1 for more details.

**Table 1:** Patient demographics and characteristics.

		Group A	Group B	*t* value	Chi-square value	*p*-value
Age		57.7 ± 10.7	54.2 ± 11.3	1.093		0.777
Sex	Man	9	14		2.087	0.149
	Woman	15	10			
BMI		23.0 ± 3.2	23.2 ± 3.0	0.185		0.664
ASA	I	0	1		3.276	0.194
	II	21	16			
	III	3	7			
ARISCAT scores		47.6 ± 5.3	48.0 ± 5.7	0.229		0.820
Operation time (min)		622.3 ± 133.3	620.0 ± 210.0	0.045		0.964
Anesthesia time (min)		723.8 ± 124.6	710.0 ± 221.7	0.262		0.795
Crystal liquid (mL)		6489.6 ± 2360.2	6750.0 ± 2830.4	0.339		0.736
Colloidal liquid (mL)		1215.9 ± 609.0	1154.3 ± 860.2	0.271		0.789
Plasma (mL)		771.7 ± 228.8	776.7 ± 268.7	0.068		0.946
Blood (mL)		887.5 ± 744.04	620.8 ± 379.7	1.531		1.326
RBC (U)		4.88 ± 2.88	3.4 ± 1.9	1.884		0.067
NE (mg)		741.38 ± 519.93	862.5 ± 363.4	0.2436		0.8097
Urine (mL)		2727.1 ± 1387.5	2,250 ± 1279.9	1.212		0.2371
Total input (mL)		8753.8 ± 2637.3	9541.7 ± 3,755	0.824		0.415
Total output (mL)		4035.4 ± 2272.5	2943.5 ± 1164.1	1.833		0.074

*t*-test was used for age and BMI, and chi-square test was used for gender. Data are shown as mean ± standard deviation. ^*^p < 0.05 indicates a statistically significant difference. BMI, body mass index; NE, infusion of vasoactive drugs.

### The optimal PEEP level

**Figure 3:**
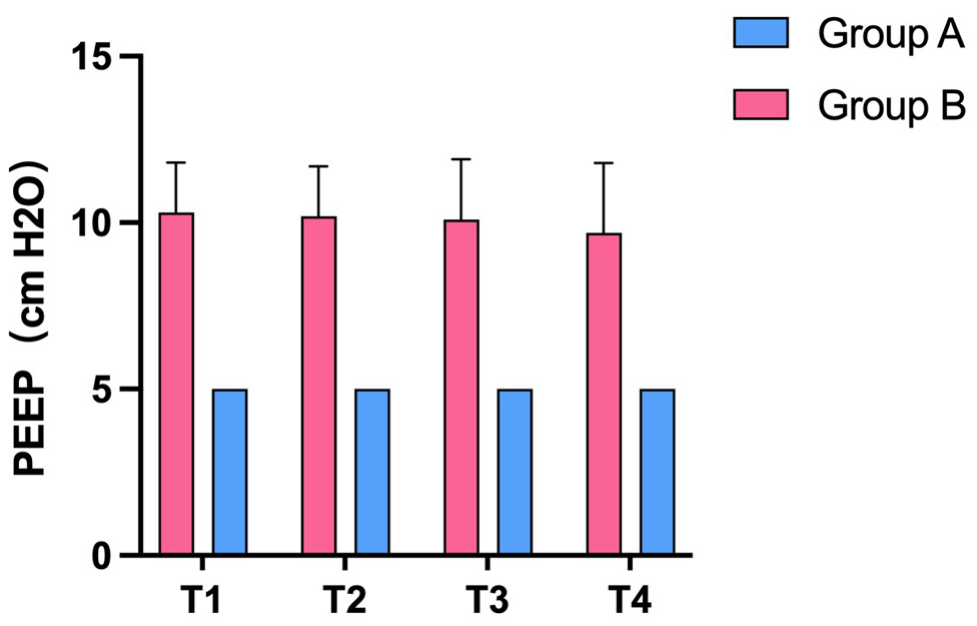
The average value of individualized PEEP in the experimental and control groups at different time periods. Statistic analysis. All data were expressed as mean ± standard error.

**Figure 4:**
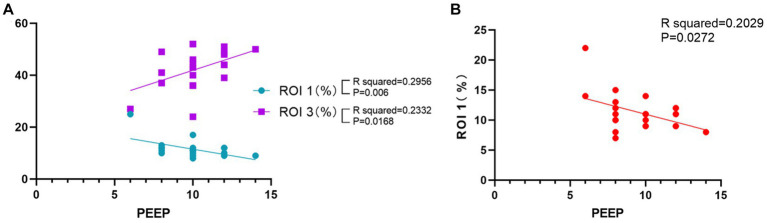
Correlation of individualized PEEP with regions of interest at different times. **(A)** Correlation of PEEP at T3 time with ROIs 1 and 3. **(B)** Correlation of PEEP at T4 time with ROI 1. The results shown were all statistically significant (^*^p < 0.05).

We analyzed the average individualized PEEP values of patients in group B at each time point. The average individualized PEEP at T1 time was 10.3 ± 1.5 cm H_2_O, T2 time was 10.2 ± 1.6 cm H_2_O, T3 time was 10.1 ± 1.8 cm H_2_O, and T4 time was 9.7 ± 2.1 cm H_2_O. Average values are shown in Figure 3. We also analyzed the relationship between individualized PEEP and ventilation in each ROI at different time periods. As shown in Figure 4A, at T3, individualized PEEP was negatively correlated with ROI 1 (*R* squared = 0.2956; *p* = 0.006) and positively correlated with ROI 3 (*R* squared = 0.2332; *p* = 0.0168) at T3, which suggested that the higher level of PEEP was associated with better dorsal lung ventilation at 4 h mechanical ventilation. In Figure 4B, individualized PEEP was negatively correlated with ROI 1 ventilation at T4 (*R* squared = 0.2029; *p* = 0.0272), which also suggested that the higher level of PEEP still works with lower ventral lung ventilation at 6 h.

### Ventilation in each ROI at different time points between the two groups

**Figure 5:**
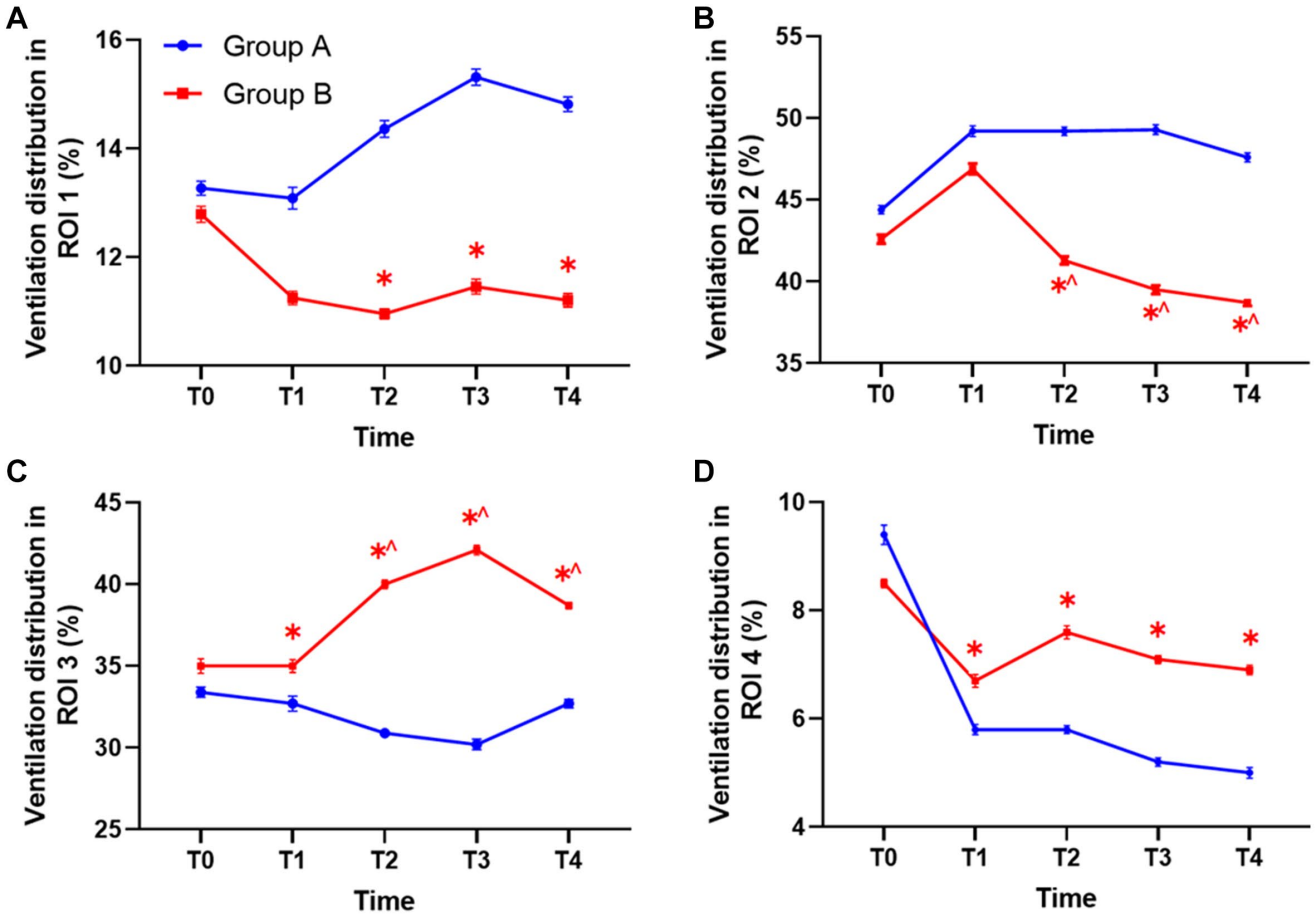
The ventilation distribution in each ROI at different time points between the two groups. **(A)** The ventilation in ROI 1 between groups. **(B)** The ventilation in ROI 2 between groups. **(C)** The ventilation in ROI 3 between groups. **(D)** The ventilation in ROI 4 between groups. Statistic analysis. All data were expressed by mean ± standard error. ROI, region of interest. (^*^p < 0.05 vs. control group, ^^^*p* < 0.05 vs. T1).

Compared with group A under PEEP 5 cm H_2_O, the percentage of ventilation distribution in the ventral non-dependent lung areas ROI 1 and ROI 2 at T2, T3, and T4 significantly decreased in group B under individualized PEEP. Moreover, ventilation distribution under individualized PEEP was higher in dorsal gravity-dependent lung areas ROIs 3 and 4 than in group A at T1 to T4 (Figures 5A–D, ^*^p < 0.05).

We also analyzed how the ventilation distribution changed with time in each group. Compared with T1, the ventilation volume was significantly lower at T2, T3, and T4 in ROI 2 of group B under individualized PEEP. Accordingly, the ventilation volume was significantly higher at T2, T3, and T4 in ROI 3 of group B under individualized PEEP (^^^*p* < 0.05) (Figures 5B,C). Taken together, these results also suggested that the ventilation distribution moved backward under individualized PEEP adjustments with time. In group A under PEEP 5 cm H_2_O, the ventilation distribution in each ROI at each time point showed no change compared with T1 after intubation. See Table 2 and Figure 5 for more detailed data.

**Table 2:** Electrical impedance tomography and respiratory parameters during mechanical ventilation.

		Times					*p*-value						
							Comparison between groups				Compared with T1		
		T0	T1	T2	T3	T4	T1	T2	T3	T4	T2	T3	T4
ROI 1 (%)	A	13.3 ± 3.1	13.1 ± 4.6	14.4 ± 3.4	15.3 ± 3.3	14.8 ± 3.0	0.116	<0.0001	<0.0001	<0.0001	0.343	0.156	0.179
	B	12.8 ± 3.5	11.3 ± 2.9	11.0 ± 2.3^*^	11.5 ± 3.3^*^	11.2 ± 3.0^*^					0.709	0.823	0.962
ROI 2 (%)	A	44.4 ± 5.6	49.2 ± 7.5	49.2 ± 5.6	49.3 ± 6.5	47.6 ± 6.1	0.335	<0.0001	<0.0001	<0.0001	0.816	0.825	0.474
	B	42.6 ± 6.8	46.9 ± 8.0	41.3 ± 5.7^*,^^	39.5 ± 6.3^*,^^	38.7 ± 4.6^*,^^					0.008	0.001	<0.0001
ROI 3 (%)	A	33.4 ± 6.9	32.7 ± 10.7	30.9 ± 6.1	30.2 ± 7.2	32.7 ± 5.5	0.448	<0.0001	<0.0001	<0.0001	0.533	0.405	0.920
	B	35.0 ± 10.6	35.0 ± 9.5^*^	40.0 ± 6.2^*,^^	42.1 ± 7.2^*,^^	43.5 ± 6.4^*,^^					0.043	0.007	0.001
ROI 4 (%)	A	9.4 ± 3.9	5.8 ± 2.1	5.8 ± 1.6	5.2 ± 1.7	5.0 ± 2.2	0.024	0.014	0.002	0.004	0.644	0.695	0.392
	B	8.5 ± 1.8	6.7 ± 2.8^*^	7.6 ± 2.9^*^	7.1 ± 2.0^*^	6.9 ± 2.1^*^					0.281	0.529	0.733
PEEP (cm H_2_O)	A	—	—	—	—	—	—	—	—	—	—	—	—
	B	—	10.3 ± 1.5	10.2 ± 1.6	10.1 ± 1.8	9.7 ± 2.1	—	—	—	—	—	—	—
CI (min·m^2^)	A	3.6 ± 1.0	2.4 ± 0.8	2.4 ± 0.8	2.5 ± 0.8	2.7 ± 0.8	0.881	0.350	0.393	0.844	>0.9999	0.848	0.933
	B	3.1 ± 0.9	2.3 ± 0.6	2.8 ± 0.6^^^	2.7 ± 0.8^^^	2.8 ± 0.8^^^					0.042	0.050	0.030
MAP (mmHg)	A	110.71 ± 20.0	102.3 ± 26.1	105.3 ± 22.1	100.7 ± 22.4	99.9 ± 25.5	0.025	0.002	0.006	0.004	0.668	0.831	0.760
	B	99.2 ± 10.8	88.67 ± 10.6^*^	87.88 ± 11.0^*^	85.08 ± 12.7^*^	82.33 ± 11.2^*^					0.805	0.306	0.055
SVV (%)	A	9.0 ± 2.7	9.7 ± 3.0	9.4 ± 4.3	9.4 ± 4.5^^^	8.0 ± 3.7^^^	0.131	0.319	0.609	0.488	0.186	0.048	0.030
	B	9.0 ± 2.7	9.7 ± 3.0	9.4 ± 4.3	9.4 ± 4.5	8.0 ± 3.7					0.820	0.824	0.107
PO_2_ (mmHg)	A	114.4 ± 68.8	239.6 ± 83.2	222.2 ± 53.3	212.6 ± 49.2	228.8 ± 67.4	0.505	0.890	0.089	0.993	0.404	0.188	0.237
	B	123.6 ± 68.9	226.6 ± 40.0	224.1 ± 38.7	233.0 ± 26.9	229.0 ± 26.8					0.831	0.532	0.817
PCO_2_ (mmHg)	A	42.8 ± 4.3	40.1 ± 4.8	38.1 ± 4.3	38.0 ± 4.0	39.0 ± 4.0	0.585	0.048	0.005	0.068	0.153	0.114	0.384
	B	40.6 ± 4.7	39.4 ± 4.3	41.0 ± 5.1^*^	41.8 ± 4.6^*^	41.3 ± 4.5					0.252	0.076	0.142
Cdyn (mL/cm H_2_O)	A	—	52.1 ± 13.0	46.5 ± 13.4	46.6 ± 9.3^^^	45.9 ± 8.1^^^	0.844	0.954	0.286	0.383	0.278	0.025	0.029
	B	—	50.9 ± 14.1	46.2 ± 11.7	51.8 ± 11.3	50.5 ± 12.6					0.221	0.809	0.908
*P*_peak_ (cm H_2_O)	A	—	17.7 ± 2.6	17.0 ± 3.5	19.3 ± 1.6^^^	18.6 ± 1.7^^^	0.004	0.001	0.057	0.067	0.358	0.004	0.009
	B	—	21.9 ± 3.1^*^	22.0 ± 2.8^*^	21.9 ± 3.2	21.2 ± 3.4					0.886	>0.9999	0.460
*P*_plat_ (cm H_2_O)	A	—	14.8 ± 1.7	15.8 ± 2.0^^^	15.3 ± 1.7^^^	15.3 ± 0.9^^^	0.037	0.178	0.121	0.216	0.045	0.001	0.000
	B	—	17.7 ± 3.0^*^	17.9 ± 3.3	17.7 ± 3.4	17.1 ± 3.2					0.860	>0.9999	0.500
*P*_mean_ (cm H_2_O)	A	—	8.6 ± 0.5	8.3 ± 0.7	8.4 ± 0.9^^^	8.7 ± 1.2^^^	0.000	0.007	0.007	0.029	0.109	0.010	0.004
	B	—	11.8 ± 2.0^*^	11.5 ± 2.5^*^	11.5 ± 2.7^*^	11.3 ± 2.8					0.666	0.719	0.446
The driving pressure (cm H_2_O)	A		8.7 ± 2.4	10.1 ± 2.2	11.1 ± 2.2	11.5 ± 2.3	<0.854	0.149	0.008	0.001	0.045	0.001	0.001
	B		8.5 ± 3.5	8.7 ± 4.0	8.5 ± 4.0	7.9 ± 4.2					0.883	>0.999	0.587

ROI, region of interest; PEEP, positive end-expiratory pressure; CI, cardiac index; SVV, Stroke volume variability; Cdyn, dynamic lung compliance; *P*_peak_, peak airway pressure; *P*_plat_, airway plateau pressure; *P*_mean_, mean airway pressure. ^^^*p* < 0.05 compared with T1, ^*^p < 0.05 compared with two groups of AB.

In the control group, group A, the driving pressure was 8.7 ± 2.4 cm H_2_O at T1, 10.1 ± 2.2 cm H_2_O at T2, 11.1 ± 2.2 cm H_2_O at T3, and 11.5 ± 2.3 cm H_2_O at T4, with a trend of increase, while in the individualized PEEP group, group B, the driving pressure was 8.5 ± 3.5 cm H_2_O at T1, 8.7 ± 4.0 cm H_2_O at T2, 8.5 ± 4.0 cm H_2_O at T3, 7.9 ± 4.2 cm H_2_O at T4, with a trend of decrease. There was a significant difference at T3 and T4 between groups, suggesting that an individualized PEEP setting was especially better for this kind of long-duration operation.

We tested the four ROIs by analyzing the ANOVA with different groups and time as two factors. The results are shown in Table 3. We found that, for ROIs 1 and 4, the difference in ROI ventilation between the two groups was only affected by the different PEEP regimens and not by time. For ROIs 2 and 3, the differences were affected by both time and PEEP regimen, and all differences were statistically significant (*p* < 0.05).

**Table 3:** For the iPEEP group and PEEP5 group differences in ventilation at different times.

Source	Partial SS	df	MS	*F*	*p*
ROI 1	569.23	7	81.318	6.92	<0.001
Group	520.083	1	520.083	44.24	<0.001
Time	27.188	3	9.063	0.77	0.512
Group#time	21.958	3	7.319	0.62	0.601
Residual	2163.25	184	11.757		
Total	2732.479	191	14.306		
ROI 2	3306.5	7	472.357	11.04	<0.001
Group	2282.521	1	2282.521	53.36	<0.001
Time	640.792	3	213.597	4.99	0.002
Group#time	383.188	3	127.729	2.99	0.0325
Residual	7871.167	184	42.778		
Total	11177.67	191	58.522		
ROI 3	4545.25	7	649.321	11.16	<0.001
Group	3485.021	1	3485.021	59.91	<0.001
Time	413.667	3	137.889	2.37	0.072
Group#time	646.563	3	215.521	3.7	0.013
Residual	10703.75	184	58.173		
Total	15,249	191	79.838		
ROI 4	128.813	7	18.402	3.18	0.003
Group	108	1	108	18.69	<0.001
Time	15.854	3	5.285	0.91	0.435
Group#time	4.958	3	1.653	0.29	0.835
Residual	1063.167	184	5.778		
Total	1191.979	191	6.241		

df, degree of freedom; MS, sum of squares; *F*, *F* value. *p* < 0.05 was considered statistically different.

### Respiratory and circulatory function indicators

**Figure 6:**
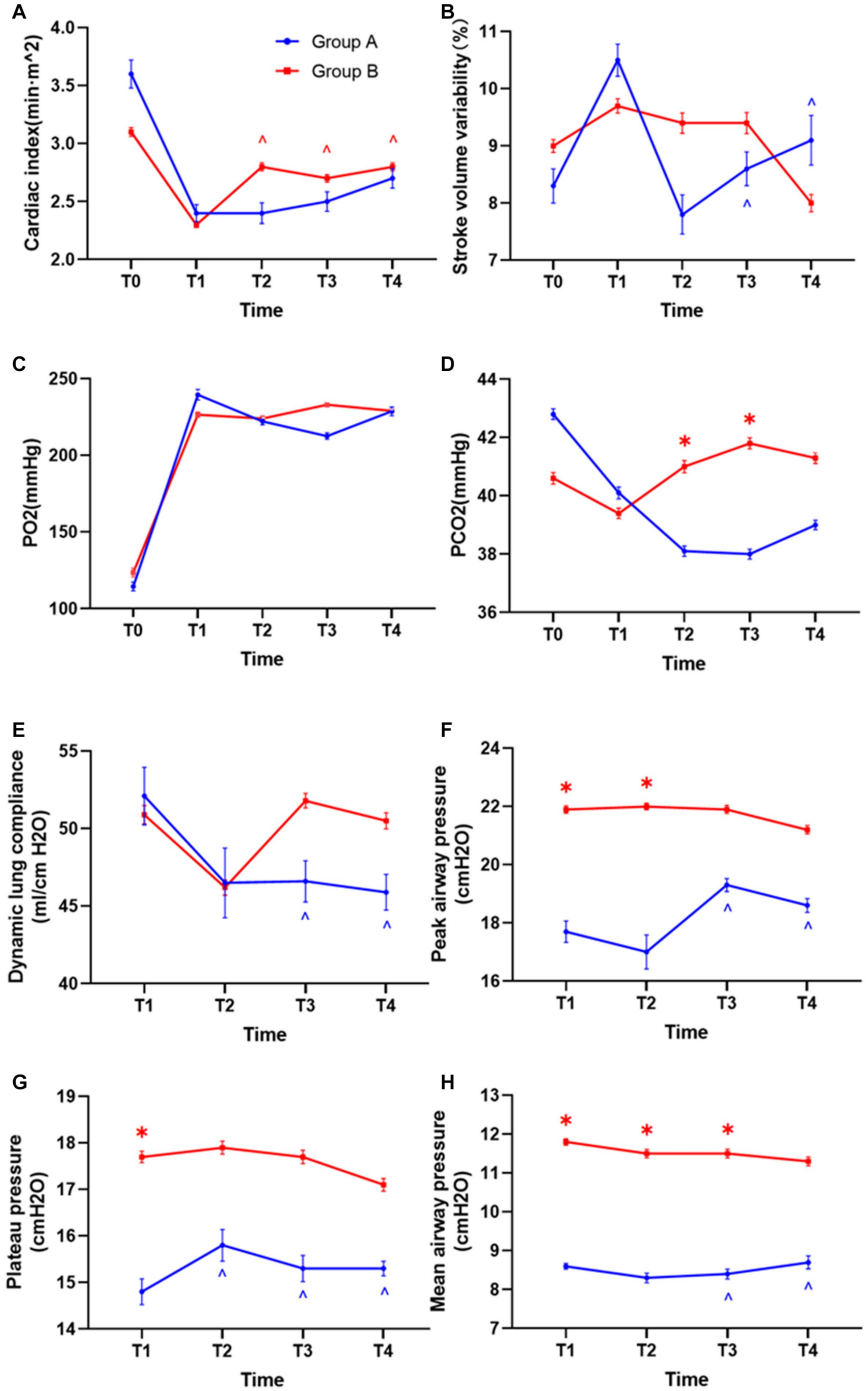
Differences in hemodynamics and respiratory mechanics between the two groups. **(A)** Differences in cardiac index between the two groups of patients. **(B)** Stroke volume variability. **(C)** Oxygen partial pressure difference. **(D)** Difference in carbon dioxide partial pressure. **(E)** Differences in lung compliance. **(F)** Differences in peak airway pressure. **(G)** Airway plateau pressure differences. **(H)** Mean airway pressure differences. (^*^p < 0.05 vs. control group, ^^^*p* < 0.05 vs. T1).

There was no significant difference in cardiac index, Stroke volume variability, partial pressure of oxygen (PO_2_), and dynamic lung compliance at any time point between the two groups (Figures 6A–C,E). The PCO_2_ of group B under individualized PEEP was significantly higher at T2 and T3 than that of group A under PEEP 5 (Figure 6D, *p* < 0.05), but it was still in the normal range. Compared with group A, the peak airway pressure at T1, T2, and T3 was significantly higher (Figure 6F, *p* < 0.05), the airway plateau pressure at T1 was significantly higher (Figure 6G, *p* < 0.05), and the average airway pressure at T1, T2, and T3 was significantly higher (Figure 6H, *p* < 0.05) in group B. However, all these indicator values in both two groups were clinically acceptable.

Accordingly, we also analyzed the changes in respiratory and circulatory functions during the mechanical ventilation maintenance period in each group. In group B under individualized PEEP, the cardiac index was significantly increased at T2, T3, and T4 compared with T1 (Figure 6A, *p* < 0.05). For all the other respiratory and circulatory function indicators, the values were kept stable in group B under individualized PEEP throughout the 6 h mechanical ventilation. However, in group A under PEEP 5 cm H_2_O, the variability in Stroke volume at T3 and T4 was significantly lower than at T1. For respiratory function, the dynamic lung compliance at T3 and T4 was statistically decreased compared to T1 under PEEP 5 cm H_2_O (Figure 6E, *p* < 0.05). Meanwhile, in group A, the peak airway pressures at T3 and T4, the plateau pressure at T2, T3, and T4, and the mean airway pressure at T3 and T4 were significantly higher than T1 (Figures 6F–H, *p* < 0.05). See Table 2 and Figure 6 for more detailed data.

### Differences in postoperative pulmonary complications between the two groups of patients

**Figure 7:**
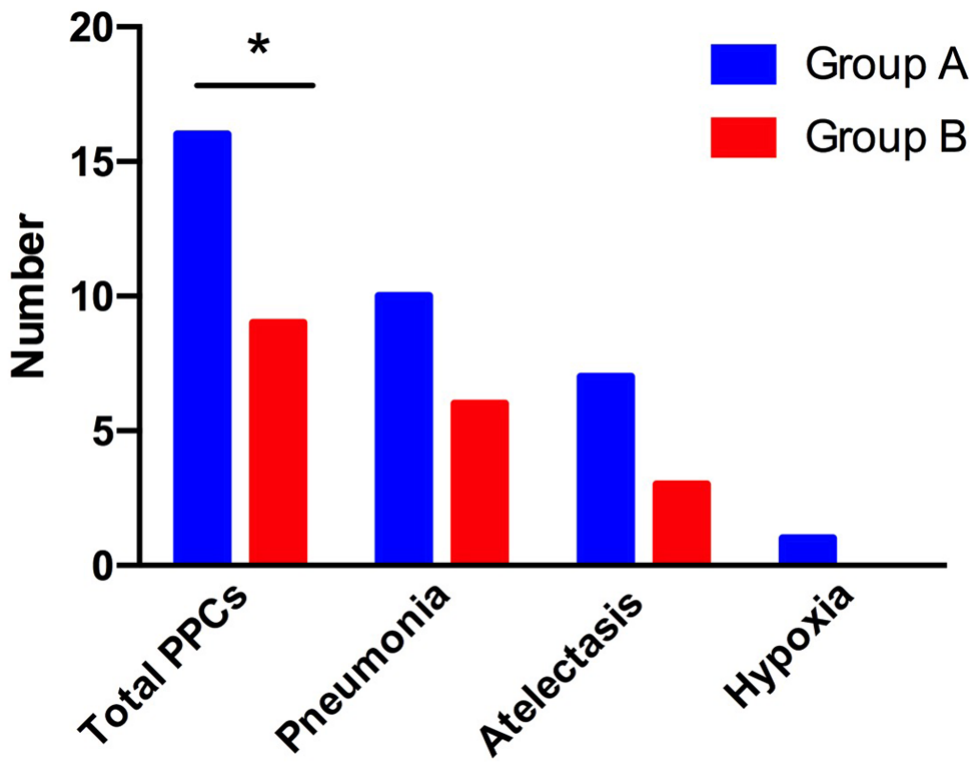
Differences in postoperative complications between the two groups of patients. The total number of patients with PPCs, pneumonia, atelectasis, and hypoxia is shown. (^*^p < 0.05 vs. control group).

We analyzed the incidence of total PPCs under individualized PEEP or PEEP 5 cm H_2_O between groups. A total of 16 patients in group A (66.7%) and 9 patients in group B (37.5%) had different types of PPCs. We conducted a chi-squared test between the two groups and found that these differences were statistically significant (chi-squared value = 4.090; *p* < 0.05). More specifically, we analyzed the patients with different kinds of PPCs: 10 patients in group A and 6 patients in group B suffered pneumonia postoperatively; 7 patients in group A and 3 patients in group B developed atelectasis; 1 patient in group A and no patient in group B suffered hypoxia. Although the difference between the two groups did not reach statistical significance, there was a considerable trend toward a decrease in the development of pneumonia and atelectasis under individualized PEEP. See Figure 7.

## Discussion

Our study showed that the optimal PEEP for patients under CRS + HIPEC was 10 cm H_2_O, which was stable during 6 h of mechanical ventilation. The individual PEEP provided ventilation distribution back-shift and reduced the incidence of total PPCs for patients.

The optimal PEEP may vary for different operations, positions, or patients. He et al. (24) suggested an individualized PEEP guided by EIT of 10 cm H_2_O for laparoscopic abdominal surgery. Similar results were also reported by Severgnini et al. (25), comparing the 10 cm H_2_O PEEP group and the 0 cm H_2_O PEEP (ZEEP) group and finding that the 10 cm H_2_O PEEP group had better postoperative lung function, had fewer postoperative complications and pulmonary infections, and had a higher rate of discharge on postoperative day 14. Studies also showed that higher PEEP pressure (15 cm H_2_O) is better than lower (5 cm H_2_O) in some special postural surgeries (23). In some clinical trials, PEEP has been set with goals for optimal oxygenation (26) or based on optimal respiratory mechanics (27). In our study, we found significant differences in ventilation distribution between the two groups. The group under the guidance of EIT showed better ventilation in the dorsal areas of the lung and kept these areas open. This results in a more uniform ventilation distribution. In several previous retrospective studies, it was found that, when conventional PEEP level of 5 cm H_2_O was used for ventilation, the lungs could not be fully ventilated during surgery in some special positions (23, 28). Therefore, individual PEEP, under the guidance of EIT, can solve some special problems. Higher recovery of dorsal lung ventilation may exhibit beneficial physiological effects in the current study.

In some previous studies (29–31), the investigators also titrated PEEP with EIT, and the results were clinically significant, but it was not used in the previous report to apply it to longer procedures for comparison. In this study, titration was performed using the stable EIT-EELI method. EIT titration of individualized PEEP showed a decreasing trend with time, but the difference was not significant. There are many ways to titrate an individualized PEEP (32). (1) The EELI method involves increasing or decreasing PEEP levels by repeatedly measuring EELI levels until the EELI levels are stable (less than 10% decrease). In addition to improving patient oxygenation, this method can be used to set PEEP in patients with severe ARDS[1] and to titrate external PEEP in patients with severe asthma (33). (2) Costa’s approach indicates that the highest PEEP levels can lead to alveolar hyperexpansion and reduced lung compliance, while the lowest PEEP levels can lead to alveolar hyperexpansion and collapse. The intersection of the EIT curves was used to determine the optimal PEEP between alveolar hyperexpansion and alveolar collapse. It has been suggested that such a PEEP value poses a threat of alveolar overdistension and alveolar collapse, especially in patients with ARDS (34). The optimal PEEP level adjusted with time was guided by EIT every 2 h. Since the atelectasis and respiratory indicators may worsen with prolonged mechanical ventilation, our results suggested that the appropriate PEEP was stable during 6 h for patients under CRS + HIPEC. The ventilation distribution moved back and forth under individualized PEEP with time. For conventional PEEP, although the ventilation distribution did not change significantly, the peak airway pressure, plateau pressure, and mean airway pressure increased with time, accompanied by decreased dynamic lung compliance, which was consistent with our perception and indicated poor lung function after major surgery under long-term mechanical ventilation. The PCO2 was 41.0 ± 5.1 mmHg in group B and 38.1 ± 4.3 mmHg in group A at T2 and 41.8 ± 4.6 mmHg in group B and 38.0 ± 4.0 mmHg in group A at T3 (Figure 6D, *p* < 0.05). The PCO_2_ was higher in group B under individualized PEEP than that in group A under PEEP of 5 cm H_2_O. The difference in PCO_2_ was approximately 3 mmHg and within the normal range, but we still could not exclude whether some lung regions were overdistended in some patients. However, the PPCs were significantly decreased under an individualized PEEP. In future studies, we will employ more indexes to analyze the changes in dead space.

PEEP can also cause circulatory dysfunction. Too high PEEP levels can increase intrapulmonary pressure, compress the cardiovascular and alveolar septa, cause hemodynamic and oxygenation disorders through mechanical compression and the neurohumoral reflex, and even cause barotrauma and malignant arrhythmias (35). The effects of PEEP on the circulatory system were evaluated in our study. High PEEP can increase the average intrathoracic pressure, obstruct the return of the superior and inferior vena cava, reduce the cardiac preload, and lead to a decrease in the cardiac index. Dambrosio et al. (36) reported that PEEP can decrease the right ventricular ejection fraction and increase the right ventricular end-systolic volume. When a PEEP of 15 cm H_2_O is used, the circulatory function of the patient can be affected (23). In our study, the CI and SVV under individualized PEEP were not significantly different from conventional PEEP, suggesting that such a PEEP level might not affect the patient’s circulatory function. Besides, the total input (crystal liquid and colloidal liquid) and the dosage of NE used were not increased under individualized PEEP.

In our study, pneumonia and atelectasis were the main types of PPCs after CRS + HIPEC. EIT-guided individualized PEEP strategies significantly reduce the incidence of total PPC compared with conventional PEEP. Additionally, the trend of pneumonia and atelectasis was considerably lower in the individualized PEEP group. Mechanical ventilation-related atelectasis may lead to lung injury. Previous studies have shown that (37) atelectasis injury is associated with forced repeated opening and closing of the distal trachea, and high-frequency oscillatory ventilation, or PEEP, can avoid or reduce atelectasis injury (38). Similar results were shown by Zhang et al. (39), who showed that individualized PEEP of approximately 10 cm H_2_O diminished the area of atelectasis and the overall severity of PPCs after open upper abdominal surgery (40). Controversial results that individualized PEEP had no effect on postoperative lung function or PPCs were also reported, which might be due to the different types of operations and risks of developing PPCs (23, 24).

The type of surgery selected for this experiment was abdominal thermal perfusion chemotherapy, which can cause pathophysiological changes in the lungs and increase pulmonary complications. The surgical approach is complex, the operation takes a long time, and the patient needs to be put on prolonged mechanical ventilation. Patients in a hyperthermic environment had an increased metabolism, increased acidic metabolites, and increased CO_2_ production, which can cause metabolic combined respiratory acidosis. Hyperthermia can also cause interstitial pulmonary edema when combined with increased abdominal pressure, increased airway pressure, decreased lung dynamic compliance, and impaired ventilation distribution (41–43). Therefore, in this experiment, pneumonia had a higher percentage of pulmonary complications. Our study found that, after titration of PEEP values using EIT, it effectively reduced postoperative complications in patients. EIT can help determine the appropriate PEEP for these types of laparotomies. Without the help of an EIT device, our findings demonstrate that PEEP levels of 10 cm H_2_O or higher seem to cause fewer postoperative pulmonary complications than those caused by 5 cm H_2_O.

Our study has certain limitations. First, we only investigated the short-term PPCs that occurred 7 days after surgery. In future studies, longer postoperative periods like 1 month or 3 months follow-ups could be included. Second, the sample size was small, and the trial was performed in a single central hospital. Therefore, the findings need to be further confirmed with a larger number of multicenter patients. Third, the patients employed had normal cardiac and lung functions, so the results could not be extrapolated to patients with preoperative cardiac or lung dysfunctions. Finally, some factors such as BMI and duration of surgery were not corrected with individualized PEEP. Owing to the small sample size and the certain inclusion range of BMI/ duration of surgery, we will perform studies in this direction in the future.

## Conclusion

This research suggested that the appropriate individualized PEEP was stable at 10 cm H_2_O for 6 h for patients under mechanical ventilation and CRS + HIPEC. EIT-guided individualized PEEP strategies provided better intraoperative ventilation distribution and lower PPC incidence than conventional PEEP.

## Data availability statement

The original contributions presented in the study are included in the article/Supplementary material, further inquiries can be directed to the corresponding authors.

## Ethics statement

The studies involving humans were approved by the ethics committee of Beijing Shijitan Hospital, Capital Medical University. The studies were conducted in accordance with the local legislation and institutional requirements. The participants provided their written informed consent to participate in this study.

## Author contributions

LX was responsible for data acquisition and collection of patient information. KY was responsible for article writing and data processing. J-JY, LL, and W-TL were responsible for proofreading. H-HM and T-ZL provided funding and guidance. All authors contributed to the article and approved the submitted version.

## Funding

This study was supported by the Beijing Municipal Administration of Hospitals’ Youth Program (QML20200102) awarded to H-HM.

## Conflict of interest

The authors declare that the research was conducted in the absence of any commercial or financial relationships that could be construed as a potential conflict of interest.

## Publisher’s note

All claims expressed in this article are solely those of the authors and do not necessarily represent those of their affiliated organizations, or those of the publisher, the editors and the reviewers. Any product that may be evaluated in this article, or claim that may be made by its manufacturer, is not guaranteed or endorsed by the publisher.

## Abbreviations

EIT: electrical impedance tomography
PPCs: postoperative pulmonary complications
CRS: cytoreductive surgery
HIPEC: hyperthermic intraperitoneal chemotherapy
BMI: body mass index
MAP: mean arterial pressure
CI: cardiac index
SVV: Stroke volume variability
FiO_2_: inspired oxygen concentration
PETCO_2_: partial pressure of carbon dioxide
ROI: region of interest
NE: PaO_2_/FiO_2_; and norepinephrine.
