# Supplementary figures and images for: Effect of individualized positive end-expiratory pressure based on electrical impedance tomography guidance on pulmonary ventilation distribution in patients who receive abdominal thermal perfusion chemotherapy
Source: Front Med (Lausanne). 2023 Sep 5;10:1198720. doi: 10.3389/fmed.2023.1198720 (PMC10507689; doi:10.3389/fmed.2023.1198720)

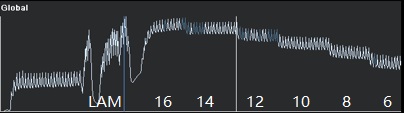

Supplement: Supplementary file 1 [file Image_1.JPEG]
